# Intraventricular continuous BDNF administration ameliorates neuroinflammation and enhances neurogenesis against rodent intracerebral hemorrhage model

**DOI:** 10.3389/fneur.2025.1606606

**Published:** 2025-08-15

**Authors:** Ting-Chun Lin, Masahito Kawabori, Erika Yoshie, Yo Nakahara, Li-Kai Tsai, Miki Fujimura

**Affiliations:** ^1^Department of Neurosurgery, Hokkaido University Graduate School of Medicine, Sapporo, Japan; ^2^Department of Neurology and Stroke Center, National Taiwan University Hospital, Taipei, Taiwan

**Keywords:** intracerebral hemorrhage, brain derived neurotrophic factor, intraventricular transplantation, inflammation, neurogenesis

## Abstract

**Objective:**

Brain-derived neurotrophic factor (BDNF) is a pivotal growth factor for neuronal survival; however, its precise role following intracerebral hemorrhage (ICH) remains poorly understood. This study sought to investigate whether intraventricular administration of BDNF could enhance neurogenesis and ameliorate neuroinflammation, resulting in improvement of neurological outcomes in a rat ICH model.

**Methods:**

Male Sprague–Dawley rats were utilized to create an acute ICH model by collagenase infusion into the internal capsule. An intraventricular minipump was subcutaneously implanted, with the catheter tip inserted into the contralateral ventricle to deliver BDNF for 7 consecutive days. The rats were assigned to one of three experimental groups; the BDNF group, the anti-BDNF antibody group, and the sham group. Functional assessment was conducted up to 14 days post-ICH, and immunohistochemical analysis was performed to evaluate neurogenesis, apoptosis, and neuroinflammation in the perihematomal area and the subventricular zone (SVZ).

**Results:**

Brain-derived neurotrophic factor treatment significantly increased the proliferation of neural stem cells in the perihematomal region. It also reduced the neuroinflammation 14 days post-ICH. Additionally, BDNF treatment demonstrated a favorable neurological function at 14 days post-ICH.

**Conclusion:**

Intraventricular administration of BDNF demonstrated favorable recovery after ICH by reducing inflammation and enhancing neurogenesis.

## 1 Introduction

Intracerebral hemorrhage (ICH) is a severe and debilitating type of stroke, with an annual incidence ranging from 16 to 36 cases per 100,000 individuals (1). Treatment methods, including surgical hematoma evacuation, have not achieved satisfactory outcomes (2, 3). highlighting the urgent need for the development of novel therapeutic modality (4).

In response to ICH, the brain initiates a series of complex remodeling processes, including neurogenesis, angiogenesis, and synaptic plasticity, to restore neurological function (5–7). The subventricular zone (SVZ) of the lateral ventricle and the subgranular zone of the hippocampal dentate gyrus are the primary regions in the adult brain where endogenous neural stem cells (NSCs) are generated (8, 9). Following an ICH, premature neuronal cells migrate to the hemorrhagic area and differentiate into neurons, enhancing neurogenesis (6, 7). However, most newborn cells fail to survive beyond 3 weeks due to apoptotic cell death. Although endogenous neurogenesis persists for at least 1 year after ICH, its insufficiency significantly hinders satisfactory functional recovery (7).

In our previous research, we demonstrated that administration of exogenous cerebrospinal fluid (CSF) derived from animals or humans after ICH enhanced neurogenesis in the primary neural stem cell cultures compared to CSF from non-ICH subjects. This improvement was attributed to brain-derived neurotrophic factor (BDNF), as higher levels of BDNF were detected in the CSF of ICH animals. Moreover, when an anti-BDNF antibody was introduced into the exogenous CSF, the enhancement effect was reversed (10). BDNF is a widely distributed intracerebral neurotrophic factor that plays a pivotal role in neuronal survival, differentiation, and the modulation of synaptic transmission and plasticity. Administering BDNF into the lateral ventricle of adult rats has been shown to induce neurogenesis including in the SVZ (11, 12). Recent studies also suggest that BDNF exhibits anti-inflammatory properties, with exogenous BDNF influencing brain inflammation via classic TrkB receptor binding and epigenetic interactions (13, 14).

In this study, the authors investigated the effects of intracerebroventricular administration of exogenous BDNF in a rat model of ICH, focusing on its impact on endogenous neurogenesis, brain inflammation, and functional recovery to evaluate its therapeutic potential against ICH.

## 2 Methods

Experimental protocols were approved by the Animal Studies Ethics Committee of the Hokkaido University Graduate School of Medicine (17–0065). All experimental procedures were conducted in accordance with the Institutional Guidelines for Animal Experimentation and the Guidelines for Proper Conduct of Animal Experiments by the Science Council of Japan. The rats were housed under controlled environmental conditions: temperature of 23 ± 3°C, humidity levels at 50 ± 20%, and a 12-h light–dark cycle. They had free access to food and water and were acclimatized to the environment for 1 week before the initiation of experiments.

### 2.1 Implantation of minipumps and administration of human recombinant BDNF or BDNF neutralizing antibody

Male Sprague–Dawley (SD) rats, aged 8 weeks and weighing between 250 and 300 g, were used in this experiment. The rats were randomly allocated to one of three groups: BDNF (*n* = 10), anti-BDNF (*n* = 10), and sham (*n* = 10). Human recombinant BDNF (AF-450–02, PeproTech; 0.672 μg in 100 μL saline) was prepared for the BDNF group, while the anti-BDNF group received a BDNF-neutralizing antibody (AB1513P, Sigma-Aldrich; 20 μg in 100 μL saline) (10, 15, 16). The sham group received saline (100 μL). All solutions were loaded into ALZET osmotic minipumps (model 1007D; Alzet, Palo Alto, CA). The rats were anesthetized with 5% isoflurane in a mixture of 30% O_2_ and 70% N_2_O, followed by maintenance anesthesia with 1.5–2% isoflurane in the same gas mixture. The rats were then placed in a small animal stereotactic frame (Model 900; David Kopf Instruments, Tujunga, CA, United States). Following a midline skin incision to expose the skull, a burr hole was drilled at coordinates 1.2 mm posterior and 2.0 mm lateral to the bregma. Then, the tip of the catheter was implanted stereotactically into the left side of the lateral ventricle (4.5 mm ventral from the skull surface). Pump was then inserted subcutaneously to the back. This would allow solution to be delivered into the brain at a constant rate of 12 μL/day for 7 days. To optimize the experimental conditions, different concentrations of recombinant BDNF (0.168 μg, 0.336 μg, and 0.672 μg) and BDNF neutralizing antibody (10 μg, 20 μg, and 30 μg) dissolved in 100 μL saline were pretested. The final experiment used the optimal concentrations of 6.72 ng/μL for recombinant BDNF and 0.2 μg/μL for the BDNF neutralizing antibody.

### 2.2 ICH model

Immediately after catheter placement, collagenase was infused to the opposite side of the brain to obtain ICH model as previously described (17). Briefly, a burr hole was drilled at coordinates 2.0 mm posterior and 3.7 mm lateral to the bregma. A 26-gauge Hamilton syringe needle was inserted 7.0 mm ventral from the skull surface. To induce the ICH, 2.5 μL of saline containing 0.2 U type IV collagenase (C5138, Sigma-Aldrich, St. Louis, MO, United States) was infused into the right internal capsule at a constant rate of 0.5 μL/min using a micro-infusion pump (Model KDS-310; Muromachi Kikai Co., Ltd., Tokyo, Japan). After the infusion, the needle was left in place for 5 min before being slowly removed, and the incision was sutured.

### 2.3 Functional outcome measurement

Functional outcomes were assessed using the modified neurological severity score (mNSS) test at 14 days post-ICH as previously described (17). One rat with a severe functional test result (mNSS = 10) was identified as an outlier and excluded from the BDNF-treated group. The final group sizes were *n* = 10 (sham), *n* = 10 (anti-BDNF), and *n* = 9 (BDNF-treated).

### 2.4 Histopathological assessment

Brain tissues were collected 14 days post-ICH for histopathological analysis. The rats were anesthetized with above mentioned condition, and then perfused with saline followed by 4% paraformaldehyde (PFA). The slices were embedded in paraffin and further sectioned into 5 μm thick slices using a manual microtome (Leica RM2125 RTS, Leica Biosystems, Nussloch, Germany). Coronal sections were selected at 4 mm posterior to the bregma, corresponding to the area with the largest hemorrhagic diameters. Immunohistochemical analysis was performed to evaluate neurogenesis, apoptosis, and inflammation in the SVZ and perihematomal areas. Neurogenesis was assessed using immunofluorescence double staining with anti-Ki67 (1:200, ab15580, Abcam) combined with anti-nestin (1:1000, ab6142, Abcam), and anti-doublecortin (DCX, 1:500, ab18723, Abcam) combined with anti-GFAP (1:500, BD 610565, BD Biosciences) (18, 19). After incubation with the primary antibodies for 1 h at room temperature, samples were treated with secondary antibodies Alexa Fluor^®^ 594 and 488 (1,200; ab150076 and ab150105 from Abcam, or R37117 from Invitrogen). Apoptotic effects were evaluated using DAB (3,3′-diaminobenzidine) immunostaining with anti-Bcl-2 (1,500, ab196495, Abcam). Anti-inflammatory effects were assessed using anti-Iba-1 (1,500, 019–19,741, Fujifilm) and anti-CD68 (1,50, MCA341GA, Bio-Rad), as Iba-1 is expressed on microglia and monocyte-derived macrophage infiltrated into brain, and CD68 is expressed on activated microglia and macrophage with high phagocytic activity. For each slide, five non-overlapping ROIs were designated in the SVZ and perihematomal areas, and were analyzed using automated cell/area counter (BZ-X700, Keyence, Japan). For perihematomal analysis, tissue sections were analyzed from n = 6 animals per group. For subventricular zone assessments, sample sizes were adjusted to *n* = 7 (sham), *n* = 9 (BDNF-treated), and n = 10 (anti-BDNF) to meet region-specific experimental requirements.

### 2.5 Statistical analysis

Data are expressed as mean ± standard error (SEM). Statistical analysis was performed using analysis of variance (ANOVA), followed by *post hoc* analysis with Tukey’s test. All statistical analyses were conducted using GraphPad Prism software (GraphPad Software, Inc., San Diego, CA, United States). Statistical significance was set at *p* < 0.05.

## 3 Results

### 3.1 Functional assessment and neuroinflammation

**Figure 1:**
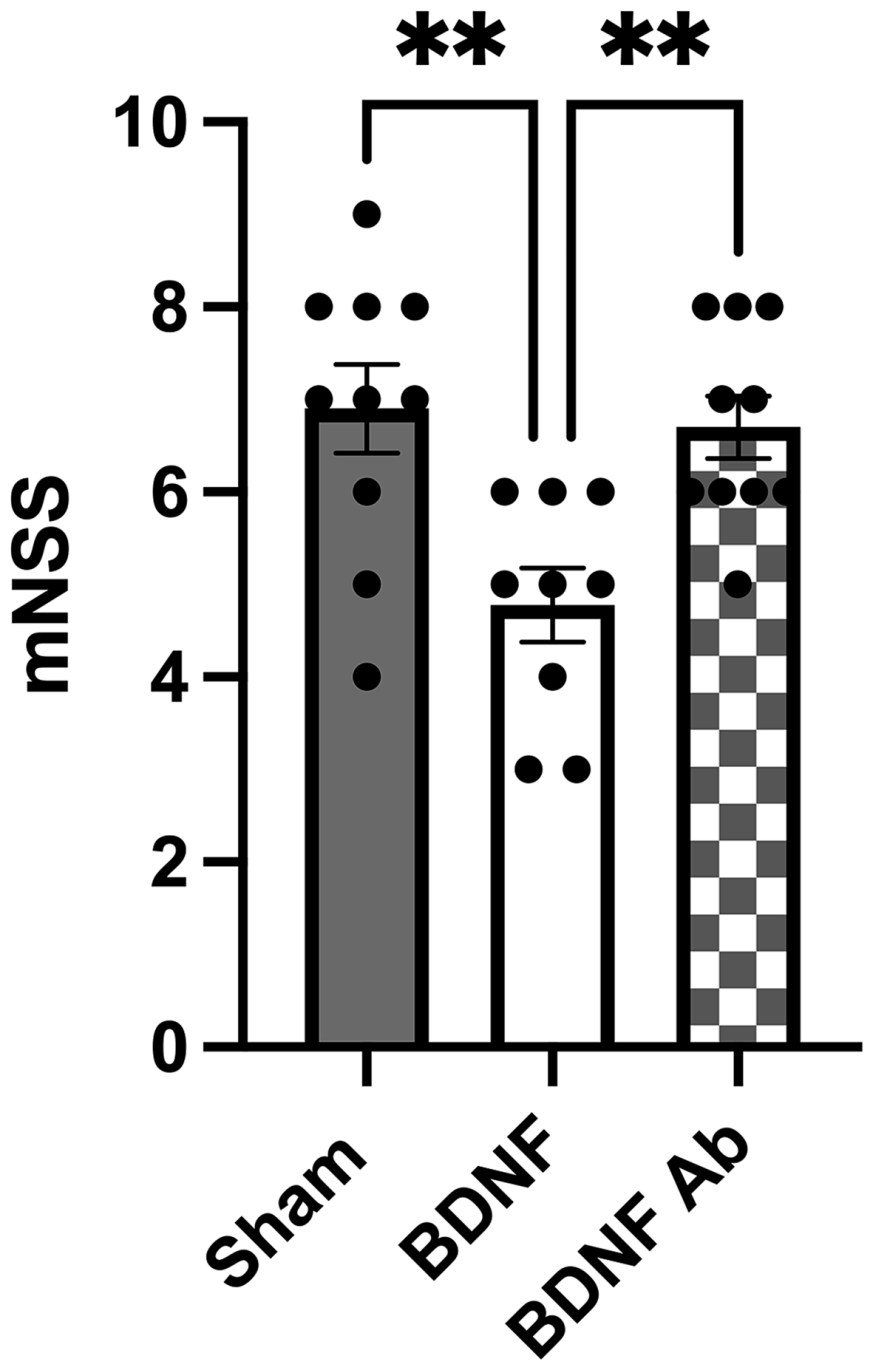
Intraventricular BDNF administration promoted functional recovery after intracerebral hemorrhage. Neurological assessment using the modified Neurological Severity Score (mNSS) at 14 days post-ICH revealed improved recovery in the BDNF-treated group (*n* = 9) compared to the sham group (intraventricular saline; *n* = 10; *p* = 0.004) and the anti-BDNF group (BDNF-neutralizing antibody; *n* = 10; *p* = 0.008). **p ≦ 0.01.

**Figure 2:**
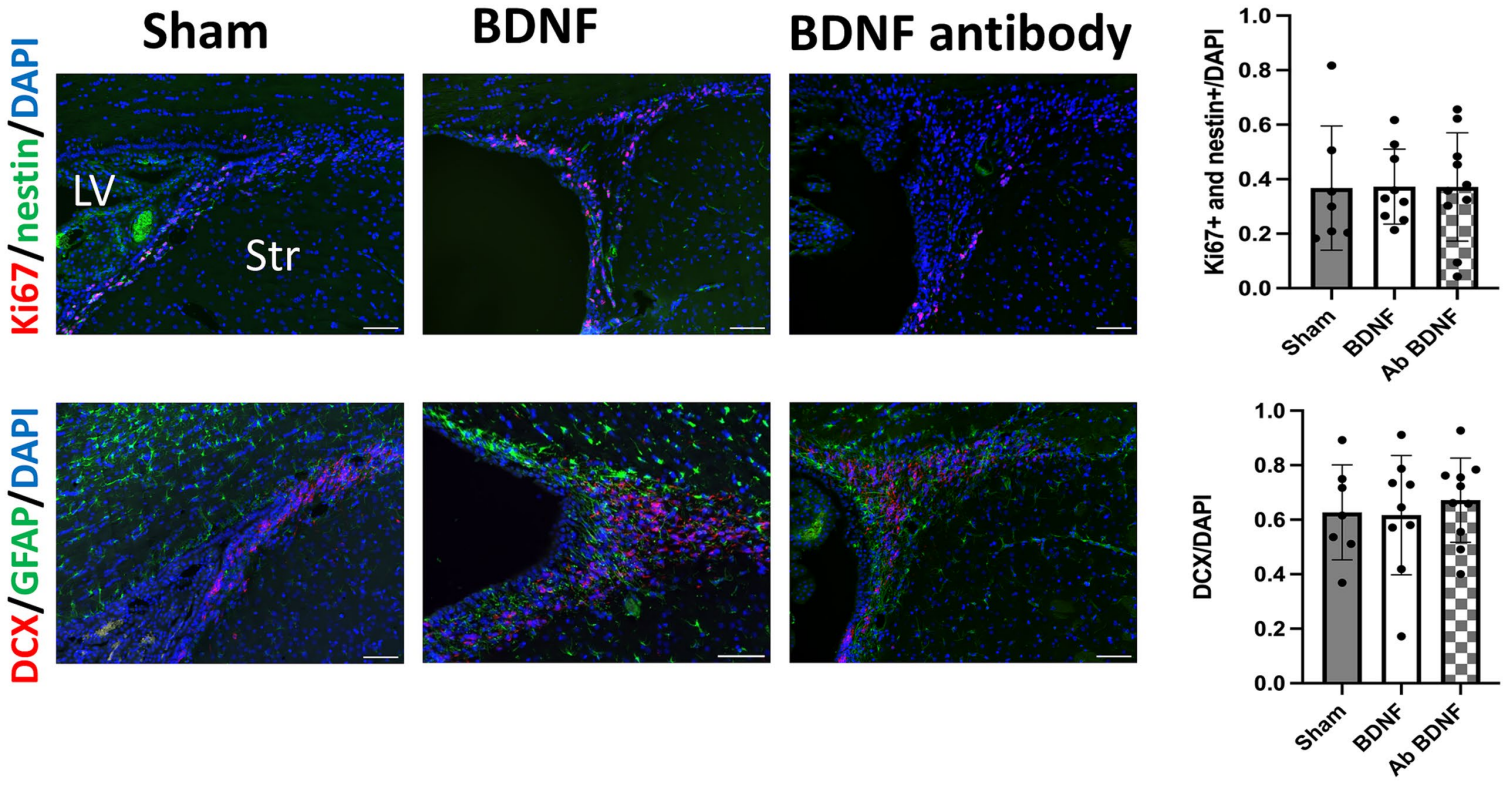
Intraventricular administration of BDNF ameliorated neuroinflammation in the perihematomal area 14 days post-ICH. The BDNF infusion group exhibited lower levels of Iba-1expression compared to the sham group (*p* = 0.09) and the BDNF ab group (*p* = 0.002) as shown in the upper row. Additionally, the BDNF infusion group demonstrated significantly reduced CD68 expression compared to the sham group (*p* = 0.02) and the BDNF ab group (*p* = 0.01) as shown in the middle row. On the otherhand, Intraventricular administration of BDNF showed no significant effect on apoptotic activity, as the percentage of Bcl-2-positive cells (an anti-apoptotic marker) showed no significant differences between the BDNF group and either the sham or anti-BDNF groups (both *p* = 0.14) (lower row) Scale bars represent 100 μm in the original image and 25 μm in the magnified image. Sample size: *n* = 6 for all groups.

Behavioral assessments using the mNSS indicated an improved functional outcome in the BDNF infusion group than the sham and anti-BDNF groups (*p* = 0.004 and 0.008, respectively) (Figure 1). The influence of BDNF on inflammation and apoptosis was further assessed. Fourteen days post-ICH, the BDNF infusion group showed no difference of Iba-1-positive cells in the perihematomal area compared to the sham group (*p* = 0.09). However, a significantly fewer Iba-1-positive cells was observed in the BDNF infusion group compared to the anti-BDNF infusion group (*p* = 0.002, Figure 2, upper row). Furthermore, the numbers of CD68-positive cells in the perihematomal area was significantly lower in the BDNF infusion group compared to both the sham group (*p* = 0.02) and the anti-BDNF group (*p* = 0.01; Figure 2, middle row). In contrast, Bcl-2-positive cells (cell survival markers; *p* = 0.14 for both comparisons; Figure 2, lower row) showed no significant differences among the three groups.

### 3.2 Neurogenesis in the SVZ and perihematomal area

**Figure 3:**
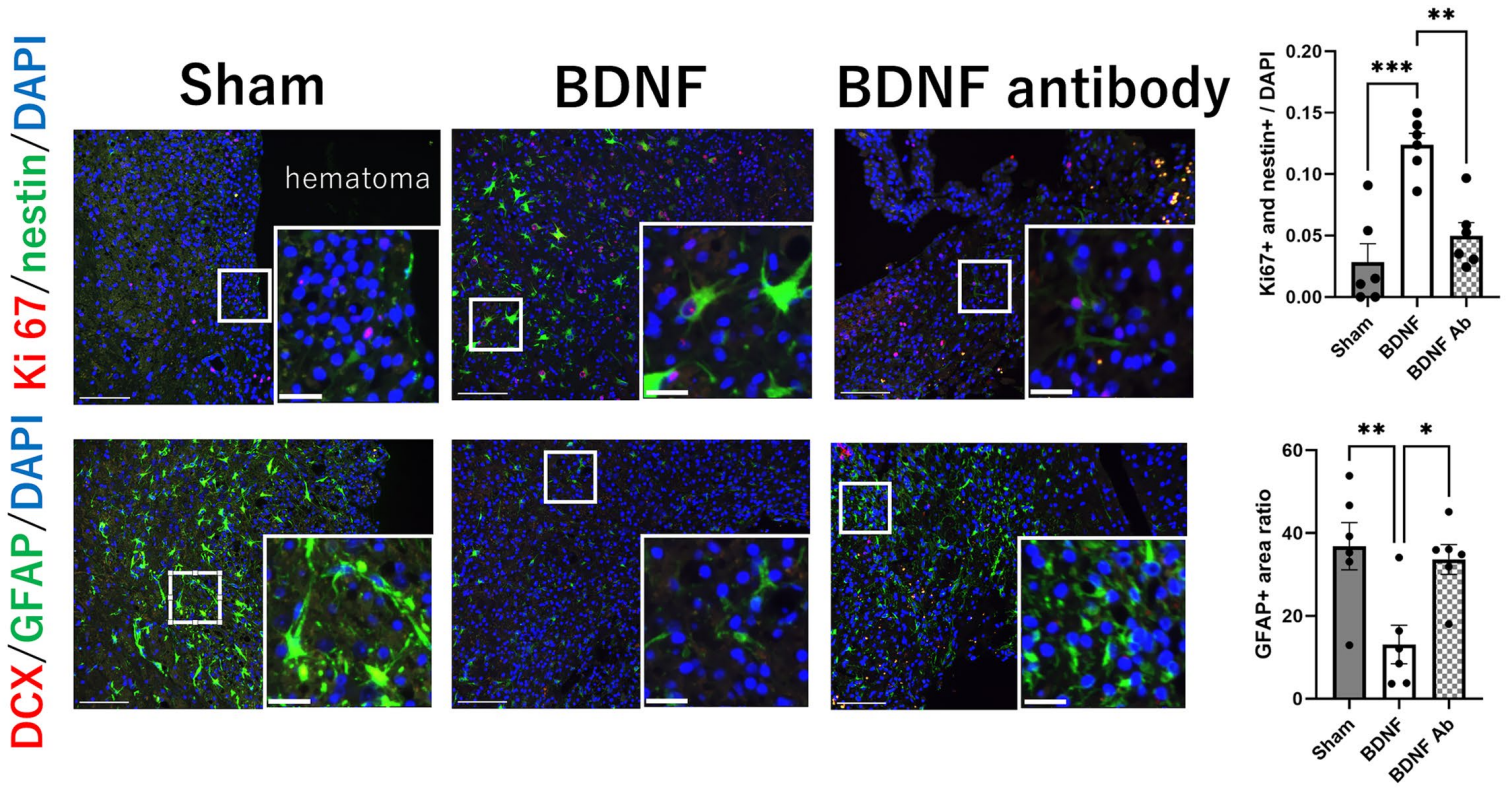
Intraventricular administration of BDNF increased the proliferation of NSCs and decreased astroglial activity in the perihematomal area 14 days post-ICH. The BDNF infusion group showed a significant increase in the percentage of proliferating NSCs, identified by Ki67 and nestin double staining, compared to the sham group (*p* = 0.0001) and the BDNF ab group (*p* = 0.0014), as shown in the upper row. Conversely, the percentage of GFAP-positive regions, indicative of astroglial activation, was significantly reduced in the BDNF group compared to the sham group (*p* = 0.0077) and the BDNF ab group (*p* = 0.0199), as shown in the lower row. Notably, the presence of DCX-positive neuroblasts in the perihematomal area was minimal across all groups. Scale bars represent 100 μm in the original image and 25 μm in the magnified image. Sample size: *n* = 6 for all groups. *p ≦ 0.05; **p ≦ 0.01; ***p ≦ 0.001.

**Figure 4:**
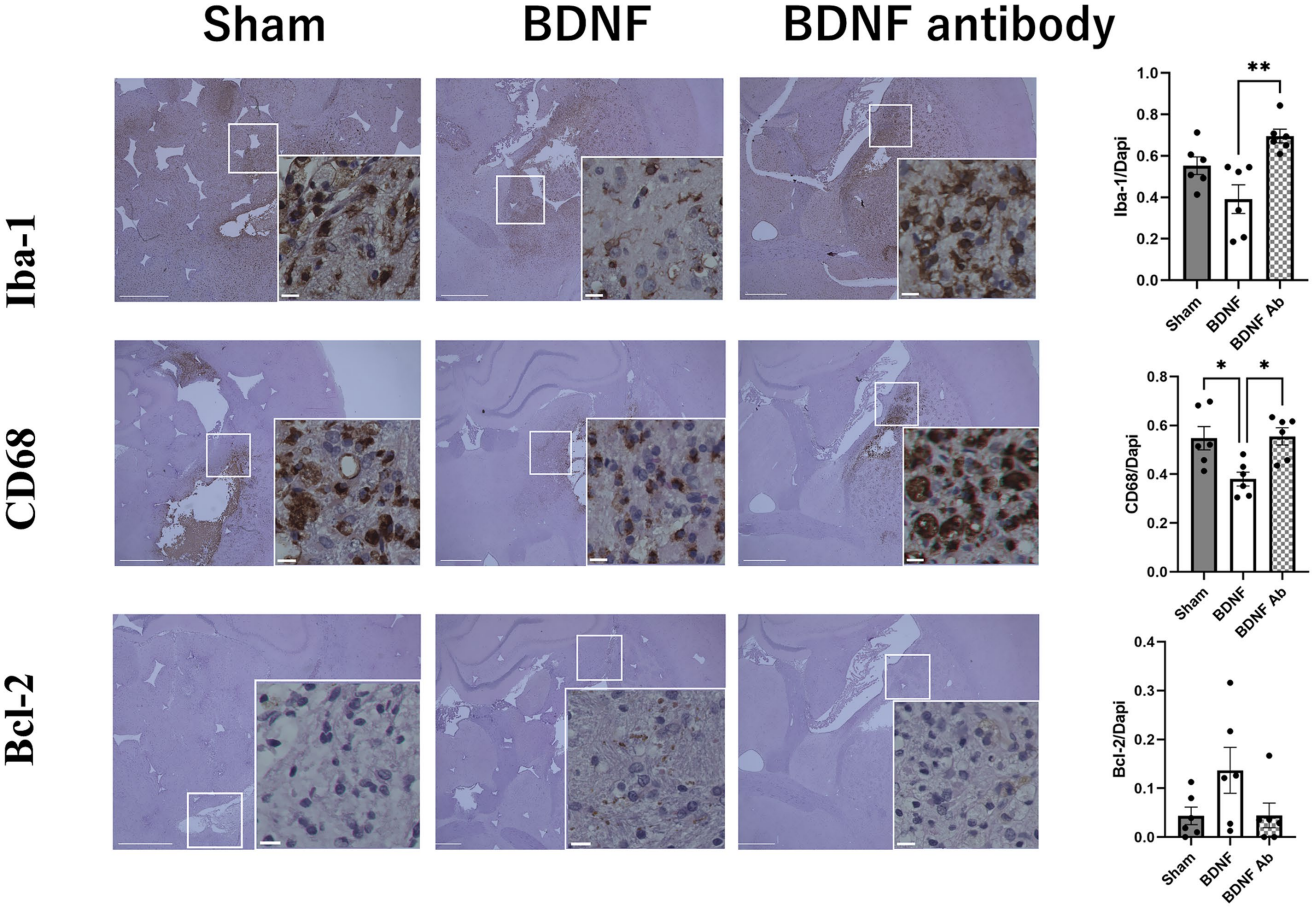
Intraventricular administration of BDNF did not increase the proliferation of NSCs or neuroblast cells in the SVZ 14 days post-ICH. The BDNF infusion group showed no significant increase in the proportion of proliferating NSCs or the density of neuroblast cells in the SVZ on the lesion side compared to the sham and BDNF ab groups (*p* = 0.99 and *p* = 0.79, respectively). Scale bars represent 100 μm. Sample size: *n* = 7 for sham, 9 for BDNF and 10 for BDNF antibody group. *p ≦ 0.05; **p ≦ 0.01.

Proliferating NSCs were identified using Ki67 and nestin double staining. The analysis revealed distinct outcomes between the perihematomal area and the SVZ. In the perihematomal area, the BDNF infusion group showed a significant increase in the percentage of proliferating NSCs, identified by Ki67 and nestin double positivity, compared to both the sham group (*p* = 0.0001) and the anti-BDNF group (*p* = 0.0014; Figure 3, upper row). Conversely, the percentage of GFAP-positive regions, indicative of astroglial activity, was significantly reduced in the perihematomal area of the BDNF infusion group than the sham and anti-BDNF groups (*p* = 0.0077 and 0.0199, respectively; Figure 3, lower row). Despite these findings, the presence of DCX-positive neuroblasts in the SVZ was minimal across all groups. Analysis revealed that the proportion of proliferating NSCs in the SVZ on the lesion side showed no significant differences when compared to the overall cell population in the SVZ across all three groups (*p* = 0.99; Figure 4 upper row). Similarly, the density of neuroblast cells, through DCX staining, in the SVZ did not vary significantly among the groups at post-ICH day 14 (*p* = 0.79; Figure 4 lower row). These data suggests that BDNF not only exerts anti-inflammatory effects but also promotes neurogenesis around the perihematomal lesion contributing to improved functional outcomes post-ICH.

## 4 Discussion

Our study demonstrated that intraventricular BDNF administration improved functional recovery in a rat ICH model. Additionally, the BDNF infusion group showed a significantly fewer numbers of Iba-1 and CD68 positive cells, which are markers of inflammation. There was also a significant increase of proliferating NSCs in the area surrounding the hematoma. Based on these findings, we suggest that BDNF may enhance functional recovery by reducing inflammation and promoting neurogenesis after ICH in rats.

Studies of human post stroke neurogenesis remain rare, due to the limitations of non-invasive techniques for analyzing neurogenesis. An especially intriguing phenomenon occurs in rodent brains, where NSCs and neural progenitor cells (NPCs) within the SVZ collaborate with ependymal cells to form a distinctive pinwheel structure. This architecture features an apical process that directly contacts the CSF and plays a crucial role in regulating NSC behavior through various specific growth factors and signaling mechanisms (20). Since the CSF circulates between the lateral ventricles and directly contacts both SVZs, the simultaneous activation of NSC proliferation and differentiation to neuroblasts in the SVZs following ICH suggests the presence of certain factors in the CSF for contributing to neurogenesis (10). After intracerebroventricular injection, BDNF exited the brain into the bloodstream at a rate comparable to the reabsorption rate of cerebrospinal fluid. This suggests that BDNF in the peripheral circulation crosses the blood–brain barrier through high capacity transport system (21). A common pharmacological approach to assess neuroregenerative and neuroprotective actions involves increasing the endogenous levels of a cerebrospinal fluid peptide by injecting or infusing the agent into a lateral ventricle (22). After brain injury or in disease, BDNF is upregulated as part of the brain’s neuroprotective and regenerative response, with contributions from neurons, astrocytes, microglia, and potentially the choroid plexus. The balance and amount from each source can vary depending on the specific disease or injury context. In our study, we used intraventricular injection of BDNF to examine the immunoreactivity of several markers on the lesional side of the SVZ 14 days after ICH. We found no significant differences in the ratio of cells co-expressing Ki67 with nestin, and DCX-positive cells among the sham, BDNF infusion, and anti-BDNF groups. Similarly, the amount of Bcl-2 expression did not significantly differ among these groups at the same time point (*p* = 0.28; data not shown in the figures). However, there was a notable increase in cells co-expressing Ki67 with nestin in the perihematomal region of the striatum at 14 days post-ICH. In the same region, the expression of Iba-1 and CD68 was downregulated. Additionally, intraventricular infusion of BDNF showed a favorable outcome, as indicated by a significant decrease in the mNSS at 14 days post-ICH. These findings suggest that intraventricular BDNF may improve neurological functional outcomes through mechanisms involving neurogenesis and anti-inflammation. A previous study investigated the striatal neurogenesis and subsequent functional recovery following chronic infusion of BDNF and Epidermal Growth Factor (EGF) in an animal model of hypoxic neonatal brain injury (23). Immunohistochemical analysis demonstrated a significant increase in the number of BrdU-positive cells in the SVZ and striatum of BDNF/EGF-treated mice compared to the PBS group 2 weeks after treatment. Additionally, there was a notable increase in the number of newly generated neurons, co-stained with BrdU and βIII-tubulin, in the neostriatum of the BDNF/EGF-treated mice. The generated cells were also observed as mobilized neuroblasts, labeled with PSA-NCAM or doublecortin, in both the SVZ and the ventricular side of the neostriatum. These findings suggest that the combined infusion of BDNF and EGF promotes striatal neurogenesis and neuronal migration in the neonatal brain following hypoxic injury, highlighting the potential therapeutic implications of this treatment approach.

Similarly, Pencea et al. (24) examined the phenotype and distribution of newly generated cells in the rat brain 16 days following intraventricular administration of BDNF, as assessed by the cell proliferation marker BrdU. The infusion of BDNF induced the proliferation of BrdU-positive cells, not only within the subventricular zone (SVZ) lining the infused lateral ventricle, but also in distinct parenchymal regions surrounding the lateral and third ventricles, including the striatum, septum, thalamus, and hypothalamus. This aligns with our finding that newly generated NSCs were located in the striatum in the perihematomal area. However, unlike other studies, we did not observe newly generated NSCs and neuroblasts in the SVZ, nor did we see migration of neuroblasts to the injured area. Other research groups have attempted to overexpress BDNF in the SVZ of rats and mice but were unable to detect a significant increase in new neurons (25, 26). Notably, one group observed an increase in BrdU-labeled cells in the rostral migratory stream (25), while another reported a slight increase in new olfactory bulb cells in the short term, followed by a significant decrease in the long term (26). These discrepancies suggest that the effects of BDNF on the SVZ may vary over the duration of infusion. It is possible that BDNF initially promotes new neuron production by accelerating progenitor differentiation and/or favoring a neuronal lineage over a glial one. However, over time, this increase in neurogenesis may deplete the progenitor pool in the SVZ, leading to a reduction in SVZ neuron production in the long term (27). Overall, the conflicting results from these studies underscore the complexity of BDNF’s effects on neurogenesis in the SVZ and highlight the need for further investigation to elucidate the underlying mechanisms and potential long-term consequences of BDNF infusion. This partially explains our finding that intracerebroventricular injection of BDNF did not lead to a significant increase in the proliferation of NSCs or the differentiation of neuroblasts in the SVZ. We propose that the observed discrepancy between our results and those of previous studies may be attributed to several factors:

1. Variations in SVZ Analysis: Our study specifically focused on the anterior portion of the dorsal lateral SVZ in rats, which may differ from the regions analyzed in previous research (24). The heterogeneity within the SVZ and differences in the subregions examined could contribute to variations in outcomes.

2. Temporal Dynamics of Neurogenesis: Our previous investigation in post-ICH rats revealed a temporal dynamic change in the proliferation of NSCs within the SVZ. We observed an increase in the percentage of proliferating NSCs, as indicated by Ki67, nestin, and DAPI triple staining, on post-ICH day 7 in the lesional side of the SVZ (10). However, the intraventricular injection of BDNF at 14 days post-ICH did not result in detectable neurogenesis in the SVZ. This suggests that the timing of the injury may influence the expression of neurogenesis. The lack of observed neurogenesis in the SVZ at the 14-day time point highlights the importance of considering the temporal dynamics of cellular processes in response to injury and treatment interventions.

Interestingly, the expression of GFAP in the perihematomal region was also decreased in the BDNF-treated group at 14 days post-ICH. It has been shown that exposure to adult human CSF potentiates NSCs to differentiate into neurons rather than astrocytes (20). Additionally, a previous study demonstrated that treatment with BDNF alone, without epidermal growth factor, resulted in an increase in neuronal marker expression and a decrease in the glial phenotype in neural stem cell culture (28). Collectively, these findings demonstrate that the adult brain parenchyma has the ability to recruit and/or generate new neurons in response to BDNF administration. These findings have important implications for potential therapeutic strategies aimed at replacing lost neurons in the adult brain following injury or disease.

Despite the absence of detectable neurogenesis in the SVZ following intraventricular injection of BDNF, our functional tests indicated better outcomes in BDNF-treated rats, as shown by improved mNSS scores. This aligns with a previous study that observed a trend of increased performance on the rotarod test 2 weeks after ischemic–hypoxic injury, with significant improvement noted at 6 weeks postoperatively (23). Takamiya et al. (17) reported that mesenchymal stem cells compounded with a biodegradable scaffold, known as CellSaic, released higher levels of trophic factors, including BDNF and extracellular vesicles, which led to improved cell engraftment and neuronal integrity. This resulted in better functional recovery starting 2 weeks after ICH, which was reversed by neutralizing BDNF (17). Moreover, we found that BDNF treatment notably reduced the inflammatory markers Iba-1 and CD68 expression. Previous research has demonstrated that intranasal BDNF may positively impact the inflammatory response following a stroke. Specifically, it was found that intranasal BDNF can decrease both protein and mRNA levels of the tumor necrosis factor-*α* (TNF-α); pro-inflammatory cytokine, while increasing levels of IL-10; the anti-inflammatory cytokine, and its mRNA. This suggests that the protective effect of intranasal BDNF in ischemic stroke might be partially due to the downregulation of TNF-α and the upregulation of IL-10. Upon detection of blood components within the parenchyma, an immediate inflammatory response is initiated, characterized by the mobilization and activation of inflammatory cells. Microglia and astrocytes are believed to be the first inflammatory cells to react against extravasated blood (29). The activation of microglia facilitates the infiltration of various circulating immune cells, particularly macrophages and T cells. Microglia also contribute significantly to the development of pathological neuroinflammation by releasing pro-inflammatory cytokines, thereby exacerbating neurotoxicity. A key mediator in the inflammatory activation process is NF-κB, a transcription factor that regulates the expression of several pro- and anti-apoptotic genes, including BDNF (30). NF-κB governs critical processes such as apoptosis, neuronal survival and proliferation, and the migration and maturation of immune cells. BDNF-induced NF-κB expression activates PLC-*γ*/PKC signaling through the phosphorylation of kinases IKKα and IKKβ. These kinases subsequently degrade IκBα, liberating NF-κB to induce the expression of genes associated with neuronal proliferation, survival, and the inflammatory response (31). In our study, levels of Iba-1, CD68, and GFAP were significantly reduced in rats receiving BDNF compared to the sham and anti-BDNF groups. This suggests that the beneficial effects of BDNF may not be directly attributable to neurogenesis within the SVZ but rather to other mechanisms, such as the modulation of neuroinflammation. These findings underscore the complexity of neurogenic processes in response to injury and therapeutic interventions. Further investigations are warranted to elucidate the mechanisms underlying the observed effects and to optimize treatment strategies for neurological conditions.

This study has several limitations. First, the size of the hematoma in the striatum after ICH was not measured, which could influence the analysis. However, the average functional score on the first day after ICH was similar across all groups (*p* = 0.56), suggesting a consistent model for evaluating recovery. Second, the study only examined outcomes at 14 days post-ICH, focusing on the acute phase. A longer-term study is needed to assess outcome changes over time. Third, BDNF activity and signaling are influenced by sex hormones, particularly estrogen, which can increase the expression of BDNF and its receptor TrkB. This hormonal regulation results in differences in BDNF function and potentially neurogenesis between males and females. However, most previous studies in this field have traditionally used male rats to minimize variability caused by hormonal cycles in females. Our experiment focuses specifically on neurogenesis following ICH, and investigating sex differences is beyond the scope of this study. It is important to note that documented differences exist between male and female rats in BDNF expression and its effects on neurogenesis, primarily due to variations in baseline BDNF levels and hormonal modulation. These differences should be taken into account when interpreting results or designing future experiments involving BDNF and neurogenesis. Fourth, our data have limitations in addressing whether intraventricular BDNF affects endogenous BDNF expression, which may be an important factor influencing BDNF’s neuroprotective effects. In our previous work, we quantitatively compared endogenous BDNF (precursor and mature forms) in the striatum and cortex between sham and post-ICH day 7 rats using western blot (10). No significant difference was observed. Additionally, we noted increased CSF BDNF at post-ICH day 7 compared to sham. Additionally, we noted increased CSF BDNF at post-ICH day 7 compared to sham (10). Accordingly, our data primarily address the effects of exogenous BDNF on the bilateral SVZ. Lastly, an outlier was excluded from the BDNF-treated group, which may introduce a Type I error. We should cautiously interpret the beneficial effects of BDNF treatment based on our results, and further large-scale studies may be needed to support this notion.

## 5 Conclusion

In this study, intraventricular administration of BDNF in rats showed improved functional outcomes following intracerebral hemorrhage. The BDNF treatment significantly increased the percentage of proliferating neural stem cells in the perihematomal area and reduced the presence of Iba-1 and CD68 positive cells, markers of inflammation, 14 days post-ICH. These findings suggest that BDNF may enhance functional recovery through promoting neurogenesis and exerting anti-inflammatory effects after ICH in rats.

Our findings suggest that intraventricular BDNF treatment may improve functional recovery following ICH in rats. Notably, the infused BDNF not only enhanced the proliferation of NSCs in the perihematomal region but also promoted anti-inflammatory responses after the occurrence of ICH.

## Data Availability

The original contributions presented in the study are included in the article/supplementary material, further inquiries can be directed to the corresponding author.
